# Retraction: The Design, Characterizations, and Tumor Angiogenesis Inhibition of a Multi-Epitope Peptibody With bFGF/VEGFA

**DOI:** 10.3389/fonc.2020.644690

**Published:** 2021-01-15

**Authors:** Frontiers Editorial Office

Following publication, concerns were raised regarding the integrity of Figures 3B and 3D. An investigation was conducted in accordance with Frontiers guidelines and the authors failed to provide a satisfactory explanation.

This retraction was approved by the Chief Editors of Frontiers in Oncology and the Editor-in-Chief of Frontiers.

